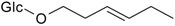# Kazakh *Ziziphora* Species as Sources of Bioactive Substances

**DOI:** 10.3390/molecules21070826

**Published:** 2016-06-25

**Authors:** Karel Šmejkal, Milan Malaník, Karlygash Zhaparkulova, Zuriyadda Sakipova, Liliya Ibragimova, Galya Ibadullaeva, Milan Žemlička

**Affiliations:** 1 Department of Natural Drugs, Faculty of Pharmacy, University of Veterinary and Pharmaceutical Sciences Brno, Brno 61242, Czech Republic; milan.malanik@seznam.cz; 2 Department of Pharmaceutical Technology, Faculty of Pharmacy, Kazakh National Medical University, Almaty 050000, Kazakhstan; zhaparkulovakarlygash@mail.ru (K.Z.); sakipova.zb@gmail.com (Z.S.); ibadullaeva.g@kaznmu.kz (L.I.); ibragimova.l@kaznmu.kz (G.I.); 3 Department of Pharmacognosy and Botany, The University of Veterinary Medicine and Pharmacy in Košice, Košice 04181, Slovakia; zemlickam1@gmail.com

**Keywords:** *Ziziphora*, essential oil, flavonoid, triterpene, cardiovascular, antibacterial

## Abstract

*Ziziphora* species represent the prototypical example of the Lamiaceae family. The phytochemicals present in *Ziziphora* include monoterpenic essential oils, triterpenes and phenolic substances belonging to the flavonoids. In Kazakh traditional medicine, *Ziziphora* species possess several medicinal uses. In particular, *Z. bungeana* Lam. and *Z. clinopodioides* Lam. are used for the treatment of illnesses related to the cardiovascular system or to combat different infections. Unfortunately, the majority of the information about the complex *Ziziphora* species is only available in Russian and Chinese language, therefore, we decided gather all available information on Kazakhstan *Ziziphora*, namely its content compounds, medicinal uses and published patents, to draw the attention of scientists to this very interesting plant with high medicinal potential.

## 1. Introduction

### 1.1. Taxonomy of Ziziphora spp. and Their Typical Habitat

Taxonomy of *Ziziphora* spp. is complicated, as its world population is represented by more than 30 different species. This genus belongs to a very large Lamiaceae family with very similar taxonomic signs. In the flora of Kazakhstan, this genus can be subdivided into six species: *Z. bungeana* Lam., *Z. clinopodioides* Lam., *Z. interrupta* Juz., *Z. pamiroalaica* Juz., *Z. tenuior* L., and *Z. vichodceviana* Tkatsch. ex Tuylaganova. It is not completely clear if *Z. bungeana* is not simply a subspecies derived from *Z. clinopodioides* [1].

*Ziziphora* plants are annual or perennial and herbaceous or sub-shrubby. Their leaves are short petiolate or sub-sessile; the leaf blade is abaxially glandular. Verticillasters are scattered on the leaf axils or crowded in a terminal capitulum; floral leaves occur as large as stem leaves or can be reduced. *Ziziphora* species blossom from June to September according to the surrounding conditions. The calyx of *Ziziphora* plants appears to be narrowly cylindric, straight to slightly curved, 13‑veined, villous, annulated at throat, obscurely 2‑lipped, with the upper lip 3‑toothed and lower lip 2‑toothed; the teeth are subequal, close together, rarely divergent after anthesis. The corolla limb of the flower is 2‑lipped: upper lip straight, margin entire, apex emarginate; lower lip spreading, 3‑lobed, and middle lobe narrower than suborbicular lateral lobes. The anterior stamens are fertile, reaching the upper corolla lip, and posterior stamens are rudimentary, short, or absent; anther cells are linear, with only 1 or 2 of them developed, and the others tend to be reduced to an appendage or absent. The style apex is unequally 2‑cleft, and the posterior lobe is short. The fruits are ovoid and smooth nutlets. As mentioned, around the world, there are about 25–30 species in Africa, Asia and Europe, and four different species in China. Kazakhstan flora is represented by six different species. The morphology of *Z. bungeana* and *Z. clinopodioides* is described in detail, and information about the phytochemically less explored species *Z. tenuior*, *Z. pamiroalaica*, *Z. vichodceviana* and *Z. interupta* can be found here [2,3].

*Z. bungeana* Lam. are aromatic subshrubs with woody roots. The stems are numerous, obliquely ascending to sub-erect, 12–30 cm long, woody at the base, branched, densely retrorse, pubescent, especially at apex. The petioles are pubescent; the leaves are narrowly lanceolate to ovate-lanceolate, rarely ovate, 5–15 mm × 1.5–6 mm, sub-glabrous or pubescent, conspicuously glandular, base cuneate to attenuate, margin entire, apex acute to slightly obtuse. The verticillasters are crowded in globose or semiglobose terminal capitula; the floral leaves are reduced, mostly ascending or horizontal. The pedicel is 1–3 mm long. The calyx is tubular, 5(–7) mm, obscurely glandular; the teeth are subequal and acute. The corolla rose, ca. 8 mm, tube pubescent, and lateral lobes are circular. Usually, 2 stamens are fertile, and the posterior stamens are short or absent. The flowering period is typically in Aug-Sep. *Z. bungeana* grows in gravelly hillsides, semi-desert areas, or sandy beaches, at altitudes of 700–1100 m above sea level in the areas of Xinjiang (China), Kazakhstan, Kyrgyzstan, Mongolia, Russia, Tajikistan, Turkmenistan, and Uzbekistan [2].

*Z. clinopodioides* Lam. has a thick and woody rhizome. The stems are numerous, simple, erect, 8–40 cm long, rarely branched, but branching on the top, somewhat ascending, covered with short hairs bent down. The leaves are petiolate, ovate or oblong-ovate, 10–25 mm long, 3–10 mm wide, with entire or unclearly rarely toothed margin. The leaves are point-ferruginous, smooth or sparsely short-haired. The flowers are gathered in dense apical capitate inflorescences surrounded by small bracts. The calyx is covered with short hairs, corolla is 10–12 mm long, pink or light purple outside, short and fluffy, tubular, twice as long as the limb, with the upper lip oblong to oval and notched, the middle part of the lower lip almost formy and villous, and lateral lobes spit ovate. *Z. clinopodioides* Lam. grows typically on the open rocky and gravelly slopes of hills and mountains, on rocky riverbanks, and also on the steppe meadows [2].

### 1.2. The Traditional Utilization of Ziziphora spp.

*Z. clinopodioides* is well known in Chinese traditional medicine as lip vanilla, leaflet mint or mountain mint. According to Chinese Materia Medica, it is used as a tranquilizing agent. It is also used to treat palpitations, insomnia, cold and fever, and oedema. It is usually administered orally as a decoction prepared by placing 15–18 g of the plant in boiling water to brew a tea. *Z. clinopodioides* is found to have been used in folk medicine to treat fevers and headaches in Xinjiang, China [4]. It is also a medicinal plant used in traditional Uighur medicine for many purposes, e.g., treatment of heart disease, high blood pressure, asthma, hyperhidrosis, palpitation, insomnia, edema, cough, bronchitis, lung abscess and other diseases [5,6]. *Z. clinopodioides* leaves, flowers and stems are frequently used as wild vegetables or additives in food to obtain a strong aroma and flavour [7].

*Z. clinopodioides* (known as blue mint bush in Turkish and Iranian traditional medicine) is well known for its antibacterial action [8]. *Ziziphora* species are used frequently also in Turkish and Iranian folk medicine, mainly as infusions for sedative, stomachic, carminative and other effects. Their antiseptic and wound healing effect is also well known [6,9]. In Anatolia, *Z. clinopodioides* is used as a wild vegetable or aroma and flavour adding spice. The plant is locally known as Kirnanesi and is prepared as an aromatic tea for treating gastrointestinal disorders and for its carminative, antiseptic and wound-healing properties [6]. Furthermore, it used as a culinary agent for manufacturing a special type of cheese [10,11].

*Z. tenuior* L. (known as raushangul in Kazakh language and kakuti in Persian language; kakuti-e kuhi is the Persian name of *Z. clinopodioides* according to Beikmohammadi [8]), is used in traditional medicine for treatment of fever, dysentery, uterus infection and as an analgesic. It is used also to combat different gastrointestinal disorders, especially as a carminative, or as a remedial agent for diarrhoea or nausea. The essential oils and main components pulegone (**53**), thymol (**62**), menthone/isomenthone (**44**) and piperitone (**45**) could be the compounds responsible for the above-mentioned medicinal properties [12]. *Z. tenuior* has high content of essential oil, meaning it is a good raw source of pulegone (**53**) which is widely used in the food and drug industry [13,14]. Sezik et al. found this plant (local name Chulhulva) to have hypotensive properties in their ethnopharmacological research on the medicinal plants of Uzbekistan [13].

*Z. bungeana* herb is used in Uygur medicine to prepare oral decoction which relieves respiratory distress, dizziness and other symptoms connected to cardiovascular diseases like coronary heart disease or hypertension [14].

## 2. *Ziziphora* Phytochemistry and Pharmacology

The previous investigations which were carried out on the *Ziziphora* genus with aim to elucidate the phytochemical profiles were mainly focused on its essential oil composition, and this will be discussed in the following text. In addition to essential oils, the *Ziziphora* species can be sources of flavonoids, caffeic acid derivatives, fatty acids, triterpenes and sterols. Ding et al. attempted to determine the effect of growth stage of *Z. clinopodioides* on the content and composition of essential oils, terpenoids, phenolics and flavonoids, showing that essential oil content was higher during the flowering period from non-volatile compounds and only the total flavonoid content was strongly affected by the growth stage [15]. Similar results were shown by Razmjoue and Zarei, where the essential oil content was correlated to temperature, relative moisture and height above sea level [16]. The habitat was also a factor affecting the content of the compounds present in the essential oils of *Z. clinopodioides* [17]. Moreover, different chemovars were identified during the chemical analysis of *Z. clinopodioides* and Iranian *Z. clinopodioides* ssp. *rigida*. The content of main essential oils components varied and allowed authors to classify analyzed species into pulegone/neomenthol, pulegone, pulegone/1,8-cineol, neomenthol and 1,8-cineol/terpinen-4-ol chemotypes [18,19].

Water and ethanol extracts of *Z. clinopodioides* showed no activity against several bacterial species, but some activity against COX-1 was recorded [20]. When different extracts from *Z. clinopodioides* subsp. *rigida* were tested for antibacterial activity against several Gram-positive and Gram-negative bacterial strains, only low activity was noted (with the exception of deodorized hot water and water-soluble methanol extracts against *Bacillus subtillis* and methanol and water-insoluble methanol extract against *E. coli*) [21]. However, methanol-water extracts of *Z. clinopodioides* and *Z. tenuior* showed low activity against several Gram-positive and Gram-negative microbial species in other assays [22].

The efficacy of methanolic extract obtained from *Z. clinopodioides* for treating inflammatory bowel disease was tested using the dextran sulphate-induced colitis model in mice. The parameters of inflammatory process were observed and it was found that TNF-α and NO levels were decreased, and the level of antioxidative defence was restored to almost the normal level [23]. Promising results in mice model of acetic acid-induced collitis showed also the water soluble portion of methanolic extract of *Z. clinopodioides*, however, compounds responsible for effect were probably not the components of essential oil [24]. The effect observed in this assay could be connected with antioxidant activity, as myeloperoxidase and TBARS levels were decreased by pretreatment of mice with different *Z. clinopodioides* extract concentrations. All doses of *Z. clinopodioides* showed significantly lowered score values of macroscopic and microscopic evaluations of colons, the effect of *Z. clinopodioides* at concentration of 300 mg/kg was comparable to that of prednisolone. The anti-inflammatory potential of *Ziziphora* was confirmed also by further study which showed, that ethanolic extract of *Z. tenuior* is active in induction of CD40 expression on dendritic cells and it can modulate the immunity response by affection of cytokine secretion, what at least partially explaines the traditional usage of this plant in treatment of imunity related diseases [25]. *Z. tenuior* hydroalcoholic extract showed the antinociceptive activity (against visceral pain) in acetic acid-induced writhing assay in mice [26].

The methanolic extract of *Z. clinopodioides* subsp. *rigida* showed higher DPPH scavenging effect than the essential oil and other types of extracts [21].

*Ziziphora* extracts were tested for their potential cytotoxic effect in gastric cancer AGS cell line and showed promising cytotoxic activity [27].

*Z. tenuior* methanol and ethanol extracts showed the ability to decrease the bitterness of caffeine and showed some antioxidant activity, making *Z. tenuior* a promising food additive [28]. Some experiments also showed the reducing power of the water extract of *Z. tenuior* in the process of the formulation of silver nanoparticles [29].

The following chapters present an overview of the compounds identified from Kazakhstan *Ziziphora* species and their biological effects. Table 1, Table 2, Table 3 and Table 4 outline the *Ziziphora* compounds and their activities. The information pertaining to each single compound which was isolated or identified in Kazakhstan *Ziziphora* species is presented here to attract attention to these interesting plants with several possible uses. The data in this research were collected using the Scifinder portal, Web of Knowledge and Science Direct. The search included articles published till April 2016, which are written in English (with limited number of papers in Russian language). The search was conducted using each single compound detected in *Ziziphora* as keyword. The articles that presented results of compounds added to mixtures as well as those that appeared in congress abstracts were not considered in this review.

### 2.1. Patents

There are several patents registered for the *Ziziphora* species and their application (or application of their isolated compounds) in the area of medicine. Capsules containing the mixture of dried aerial part extract *Z. bungeana* with *Artemisia rupestris* and *Arctium lappa* extracts are used to treat different viral infections of the upper respiratory tract. The patent applications also include the assays on the antipyretic activity of the extract in rabbits, anti-inflammatory activity in rats and antitussic activity in mice. The antiviral activity of the preparation was also evaluated in vitro [30].

The method for obtaining the flavonoid fraction of the *Z. bungeana* extract was also patented, combining the extraction of the *Z. bungeana* aerial part with organic solvent, with the dispersion of the extract into aqueous phase and filtration through macroporous resin, further washed with ethanol to get a flavonoid-rich extract [31]. This flavonoid fraction is believed to be useful in the treatment of cardiovascular diseases. Other patents cover the usage of *Z. bungeana* polyphenol and flavonoid fraction [32]. A flavonoid preparation from *Z. bungeana* to treat cardiovascular disorders is also patented [33].

*Z. clinopodioides* is also a component of Chinese traditional medicinal preparation for the treatment of paroxysmal supraventricular tachycardia [34]. *Z. clinopodioides* essential oil can be used as an oral spray to improve hygiene of oral cavity, suppress inflammation and suppress the growth of oral pathogenic bacteria [35]. *Z. clinopodioides* essential oil can be used in agriculture. The method for obtaining this oil and its application as an anti-fungal preparation against plant pathogenic fungus *Sclerotinia sclerotiorum* was patented [36]. The HPLC fingerprint for *Z. clinopodioides* compounds has also been developed using reversed-phase chromatography of diosmin (**7**), linarin (**8**) and pulegone (**53**) [37].

### 2.2. Phenolics

*Z. clinopodioides* was extracted with aim to obtain extracts with different polarity compounds to determine the total polyphenol and flavonoid content. As shown, phenolic substances are concentrated in the ethyl acetate extract, similar to flavonoids, phenolic acids and some other phenolics [5]. However, the analysis of the literature on the isolation of flavonoids or further phenolics from *Ziziphora* showed the presence of mainly lipophilic compounds of aglycone type (Table 1). From flavonoids, only a limited number of glycosides (diosmin (**7**) and linarin (**8**)) was isolated.

Several aglycons, which can be called as dietary aglycons (like apigenin (**1**) and luteolin (**3**)) were also obtained. Typically, from Lamiaceae plants, lipophilic methoxylated aglycons were extracted, e.g., thymonin (**4**) or acacetin (**5**). Some relatively uncommon fatty acid-substituted flavones ziziphorin A and B (**9** and **10**) were isolated from *Z. tenuior* [38]. Furthermore, several phenolic acids and their esters, like caffeic acid (**17**) and its ethyl ester (**18**) and rosmarinic acid (**19**), salicylic acid (**20**) and benzoic acid (**21**), and derivatives of benzyl alcohol were detected in *Ziziphora* species (Table 1). The bioactivity of *Ziziphora* flavonoids was studied for several single compounds; therefore, we will mention the activities in connection to possible *Ziziphora* use. Generally, flavonoids from *Ziziphora* species showed antioxidant, anti-inflammatory, venoprotective and anticancer activity. Several lipophilic flavonoids showed also antibacterial properties.

The antioxidant activity of flavonoid substances depends on the arrangement of the functionalities on the skeleton. Especially, the substitution and number of hydroxyl groups affects the antioxidant activity mediated by radical scavenging and metal ion chelation. As the substitution of *Ziziphora*-isolated flavonoids is not entirely favourable for scavenging and chelation, the antioxidant effect may be more related with suppression of ROS formation either by inhibition of enzymes or by upregulation or protection of antioxidant defences. Flavonoids contribute to ROS generation inhibition by the affection of the enzymes involved in their production, like microsomal monooxygenase, glutathione *S*-transferase, mitochondrial succinoxidase, NADH oxidase, and others. The antioxidant activity of *Z. clinopodioides* was tested by several methods (DPPH, superoxide, and hydroxyl radical scavenging activity). Given the high polyphenol and flavonoid content, the greatest activity was observed in ethyl acetate extract [5]. Monoterpenic glucoside shizonepetoside A (**83**) and simple flavonoids apigenin (**1**), luteolin (**3**) and diosmetin (**6**) showed potent inhibitory effects on NO production. The stereochemistry of monoterpenic glucosides is important for this effect according to these results [4]. Vasorelaxant activity was shown by those *Z.* *clinopodioides* extracts that had high concentration of polyphenolic substances [135]. The mechanism of its vasorelaxant action was also elucidated. The bioactivity guided separation of CH_2_Cl_2_ part of a hydroalcoholic extract of the whole plant, using an in vitro model of rat-isolated thoracic aortic rings led to isolation of several compounds, from which apigenin (**1**) and chrysin (**2**) showed the greatest activity [39]. Therefore, some structure-activity relationships can be assumed: the presence of 4′-hydroxy group of flavonoid, no methyl substitution at C-4′ and absence of continual substitution at positions 5, 6 and 7 of the flavonoid skeleton [39]. These results should be interpreted carefully, as these tests were carried out ex vivo on normal rat aortas, and differences can be observed after application of compounds or extracts to hypertonic animals or human.

In general, lipophilic flavonoids (flavonoids aglycons or methoxylated and prenylated flavonoids) are synthesized by plants as a part of defence against microbial infection; therefore, they can be used for antimicrobial therapy in humans. Lipophilic flavonoids isolated from *Ziziphora* species like chrysin (**2**), acacetin (**5**) or thymonin (**4**) have antimicrobial effects and are components of, for example, propolis, a well-known antimicrobial active material [50,61]. Antibacterial flavonoids probably possess multiple cellular targets rather than one specific site of action. One of their actions at the molecular level is to form a complex with proteins through nonspecific forces such as hydrogen bonding and hydrophobic effects as well as by covalent bond formation. Thus, their mode of antimicrobial action may be related to their ability to inactivate microbial adhesins, enzymes, cell envelope transport proteins, and others [136]. Lipophilic flavonoids can also kill microbes by causing disruption of the microbial membranes [136]. Therefore, the presence of a number of lipophilic flavonoids can contribute to overall antibacterial effect of the traditional medicinal usage of *Ziziphora* extracts.

As it is well known, inflammation is a normal biological process in response to tissue injury, microbial pathogen infection, and chemical irritation. Inflammation is initiated by migration of immune cells from the blood vessels and release of mediators at the site of damage. This process is followed by further recruitment of inflammatory cells and release of reactive oxygen and nitrogen species and pro-inflammatory cytokines to combat the cause of inflammation, and later to repair caused damage. Acute inflammatory process is rapid and self-limiting, but prolonged inflammation triggers chronic disorders. Natural products are often used to combat diseases connected with chronic inflammation [137,138]. The effectiveness of methanolic extract obtained from *Z. clinopodioides* for treating inflammatory bowel disease was tested in dextran sulphate-induced colitis model in mice. The parameters of inflammatory process were observed and it was found that the TNF-α level and NO level were decreased and level of antioxidative defence was restored to almost normal level [23]. *Ziziphora* is relatively rich in flavonoids, which can be considered responsible for the anti-inflammatory potential of this plant. Flavonoids like apigenin (**1**) [46], luteolin (**3**) [53,54], diosmin (**7**) [82], its aglycone diosmetin (**6**) [79] and linarin (**8**) [88,89] are reported to possess anti-inflammatory effects.

Caffeic acid (**17**) and its derivatives are often connected with different therapeutical applications: their potential anticancer activity was well reviewed [98], their effects on the cardiovascular system were reviewed by Fuentes and Palomo [99] and a large review on general applications of caffeic acid (**17**) was published recently [100]. Similar activities were observed for caffeic acid ethylester (**18**). This compound showed antihypertensive, antioxidant and anti-inflammatory activities that can be connected with usage of *Ziziphora* against diseases of cardiovascular system [101,102,103,104,105,106,107,108,109,110,111,112]. Similarly, rosmarinic acid (**19**) possesses various activities, also connected with civilization diseases like cardiovascular system illnesses, chronic inflammations and cancer [113,114,115,116].

### 2.3. Triterpenes and Steroids

There is not much information about triterpenes obtained from *Ziziphora* species, however, their presence is confirmed and some unpublished results showed their relatively high concentrations. The main triterpenic compounds identified till date in *Ziziphora* spp. are oleanolic acid (**26**), ursolic acid (**27**) and maslinic acid (**28**), together with daucosterol (**29**) as a representative of plant steroids. The bioactivity of all these compounds was well reviewed (with the exception of **29**) [139,140].

Oleanolic acid (**26**) and maslinic acid (**28**) are representatives of β-amyrin type of pentacyclic triterpenes with the carboxyl group at position C-17 of the triterpenic skeleton. Both these compounds are relatively abundant in nature and are active components of many plants with medicinal properties. Oleanolic (**26**) and maslinic (**28**) acids and their derivatives are often used as components in medical drugs with effect on the cardiovascular system. These compounds help combat different so-called “civilization” diseases, for example cardiovascular diseases including atherosclerosis and diabetes, and even cancer. This could be because they have anti-inflammatory and antioxidative properties, and both cytoprotective and cytotoxic activity depending on the conditions and type of cells. Albeit, their activity is relatively indistinctive, and the multiple potentials of these triterpenes makes them good candidates for semi-synthesis and synthesis of potent drugs [141]. Ursolic acid (**27**) is an α-amyrin type of triterpene, again with carboxylic function at C-17. Similarly to oleanolic (**26**) and maslinic (**28**) acid, it can be isolated from several plant species with potent medicinal properties [142]. Similar to previously mentioned triterpenic acids, it shows activities beneficial in the treatment of civilization diseases like for example cancer, cardiovascular diseases or chronic inflammations [142].

Concerning the folk usage and effects of *Ziziphora*, oleanolic acid (**26**) and maslinic (**28**) acid have been found to affect the cardiovascular system. Both these compounds work against LDL oxidation, thus showing antiatherogenic properties. Oleanolic acid (**27**) also causes vascular smooth muscle relaxation. **28** acts as a strong antioxidant and possesses hypoglycemic properties; it was found to reduce the insulin resistance in the mouse model of genetic type 2 diabetes. **26** is also a potent antioxidant interfering with the glutathione redox cycle, affecting the Fenton reaction, NADPH oxidase, Nrf2 and others [142]. Its anti-inflammatory effects are connected mainly with interaction with NF-κB, STAT3 dimerization and overall inhibition of gene expression of pro-inflammatory factors (COX, iNOS) [142].

*Ziziphora* species extracts are also connected with antibacterial effect, and the activity of ursolic acid (**27**) was proven against numerous Gram-positive and Gram-negative bacteria, including vancomycin-resistant *Enterococcus* and different *Mycobacterium tuberculosis* strains. Some antiviral, anti-parasitic and antifungal activity was also observed [142]. However, the authors of the majority of the cited papers are right in that more studies should be carried out to prove these effects in vivo in humans [144].

Daucosterol (**29**) is a natural phytosterol—a glucoside derived form β-sitosterol. As we did not find relevant information about its bioactivity, we tried to summarise its effects in Table 2. Several activities of daucosterol (**29**) are again in accordance with therapeutic potential of *Ziziphora* species observed both in folk medicine and scientific studies. The anti-inflammatory effect of daucosterol (**29**) was observed both in vitro and in vivo [152,153] and a 5-LOX inhibitory effect was observed [154]. Daucosterol (**29**) also acts as scavenger of free radicals in vitro [155] and as an antioxidant [156] and it inhibits nitric oxide production in LPS-activated RAW264.7 cells [157]. Furthermore, **29** showed antiproliferative [164,166] and cytotoxic [167] activity against different cancer cell lines; it induces apoptosis [165] and inhibits MDA-MB-231 cancer cell migration [169]. Some antimicrobial activities of **29** were observed, mainly against *E. coli*, *S. aureus* and *H. pylori*. The inhibition of *H. pylori* growth can be beneficial in the treatment of gastric ulcer lesions, because daucosterol (**29**)-mediated suppression of HCl/ethanol-induced gastric lesions was observed by Jeong et al. [177].

### 2.4. Essential Oil

The essential oils are probably the most studied part of *Ziziphora* phytochemical components (Table 3). As could be expected for Lamiaceae, these essential oils are predominantly composed of monoterpenic compounds; however, several sesquiterpenic substances and some other compounds were also identified by GC-MS analysis (see Table 3). The hydrodistillation is the commonly used method for obtaining *Ziziphora* essential oils, but the method used for obtaining *Z. tenuior* essential oil by supercritical fluid extraction (SFE) with higher yield was also published [179]. It is clear that the relative ratios of components vary according to the frequently occurring chemotypes in the family Lamiaceae, environmental factors, geographic origin and also extraction method [180,181,182]. For example, although we can see biochemical convergence among the *Z. clinopodioides* from different locations, owing to the frequent occurrence of chemotypes, different patterns in the composition of the oils are common. As mentioned above, the variability of essential oils components in *Ziziphora* species is really high and chemovars of one species can be found in very related habitats, as showed for example by Khodaverdi-Samani et al., who identified several chemovars of *Z. clinopodioides* ssp. *rigida* in limited area of southwestern Iran (Alpine type mountains). The essential oils obtained by hydro-distillation (content ranged from 0.12 to 0.98 mL/100 g of dry weight) were analyzed by GC and GC/MS to prove that the main chemical compositions were pulegone (**53**) (5.19% to 57.85%), limonene (**38**) (0.26% to 12.79%), 1,8-cineole (**72**) (0.00% to 27.4%), bornyl acetate (**69**) (0.47% to 9.37%), piperitenone (**46**) (0.70% to 9.05%) and menthol derivatives (for example **58**) [19]. Other study revealed *Z. clinopodioides* as plant rich in carvacrol (**73**) 52.7%, linalool (**66**) 15.9% and menthol (**56**) 14% [183]. Further literature survey indicated that the oils of *Ziziphora* species have been found to be rich in pulegone (**53**) and thymol (**62**), but there are also analysis showing low or no concentration of these substances in *Z. clinopodioides* essential oil [184]. The composition of *Z. clinopodioides* essential oil is strongly influenced by flowering stage [182]. Also, the composition of *Z. tenuior* and *Z. pamiroalaica* essential oil may vary strongly [185]. *Z. bungeana* and *Z. clinopodioides* were analysed by the same group [185], same as *Z. vychodceviana* [186].

Essential oils are almost always complex mixtures of numerous substances, and therefore their biological effects are often described as the result of a synergism of all molecules or they mirror major activities of molecules present at the highest concentrations [418]. Moreover, the synergistic action is beneficial because the bacteria can undergo adaptation to maintain their membrane functionality in the presence of sub-inhibitory concentrations of antibacterial compounds and the resistance can occur, but the complex action of essential oil can help suppress this resistance [419].

Therefore, only the biological activities of essential oils in their entirety or of their main compounds have usually been evaluated. There are some reports about bioactivities of *Ziziphora* essential oils, mainly connected with evaluation of antibacterial activity. Moreover, antioxidant properties and anti-inflammatory effect was evaluated using different methods. 5-LOX was inhibited by *Z. clinopodioides* esstential oil (could be due to the presence of compounds structurally related to fatty acids serving as substrate of LOX) [420].

Generally, the major compounds reflect the biophysical and biological characteristics of the parent essential oils quite well (as visible for example for *Origanum* oil and carvacrol (**73**) [421]), and the exhibition of their effects depends on their concentration [422,423]. The very complex mixture of compounds present in essential oil also strongly affects the smell, thickness, texture, colour and cell penetration [424], lipophilic or hydrophilic attraction and fixation on cell walls and membranes, and cellular distribution [418]. Therefore, it is sometimes better to analyse the activity of the entire essential oil and compare it with the activity of pure main components. However, some reports highlight the antagonism of single components of essential oil [281,425], so the information about the activity of pure compounds could be useful. As visible from Table 3, we tried to summarize all information about biological effects of compounds present in *Ziziphora* essential oils available in recent literature, but for some compounds the information is missing or it is scarce.

Reports on the essential oils of different *Ziziphora* species often discuss their antibacterial activity. The essential oils obtained from different Kazakh *Ziziphora* species are generally rich in oxygenated monoterpenes (see Table 3); their antibacterial effect can be attributed to the presence of these compounds; however, this effect is not the consequence of the presence of oxygenated monoterpenes only. *Z. clinopodioides* essential oils were found to be effective against both Gram-negative and Gram-positive bacterial species [184,426]. The presence of thymol (**62**) could be responsible for the antibacterial activity. The activity of *Z. tenuior* essential oil was lower [426]. Similar results have been presented by Salehi et al. [21], showing good activity of *Z clinopodioides* subsp. *rigida* essential oil against several bacterial strains (with the exception of insensitive *P. aeruginosa*). Thymol (**62**) and pulegone (**53**) showed at least partial responsibility for the antibacterial effects of these materials. Also, the assays carried out on *Z. clinopodioides* subsp. *bungeana* essential oils showed activity against both Gram-positive and negative bacterial species, and pulegone (**53**) and 1,8‑cineol (**72**) were assigned as compounds responsible for the effect [201]. The high concentration of pulegone (**53**) is mentioned when the antimicrobial activity of essential oils is analysed: it showed activity especially against *C. albicans* and *S. typhimurium*. *C. albicans* was found to be susceptible to pulegone (**53**), which was found to be twice as effective as nystatin [427,428].

In other test, the antibacterial activity of essential oil and methanolic extract from *Z. clinopodioides* was compared using 52 Gram-positive and Gram-negative bacterial species, with disc diffusion method [11]. Both tested materials varied in level of activity, with much higher activity of essential oil. Pulegone (**53**), limonene (**38**), and piperitone (**45**) appeared to be the most active substances; however, further information about antibacterial activity of these compounds is not abundant.

We attempted to summarise compounds found in the literature with the antibacterial effect of *Ziziphora* essential oils and, if possible, their underlying mechanisms (Table 3). Carvacrol (**73**) and thymol (**62**) are placed in the first place, as their antibacterial and antiseptic effect is well known [429]. Their synergic action has been described previously and is well reviewed [369]. Carvacrol (**73**) acts on *B. cereus* *via* depletion of intracellular ATP pool, changes the membrane potential and increase the permeability of membrane for protons and potassium. Carvacrol (**73**) integrates into the lipidic monolayer of the cell membrane, changes its fluidity and damaging its functions [430]. There are evidences of other mechanisms of antibacterial effect, such as interaction with DNA. Moreover, application of carvacrol (**73**) has been found to inhibit the formation of bacterial biofilm, which is one of the mechanisms of bacterial resistance [369].

Thymol (**62**), an aromatic *p*‑menthane type monoterpene phenol, isomeric to carvacrol (**73**), is established as a good antimicrobial agent, interacting both with outer and inner cytoplasmic cell membranes via incorporation of the polar head group region into the lipid bilayer. This interaction changes the properties of the cell membrane and leads to its increased permeability/disintegration [431,432,433]. Moreover, thymol (**62**) can also up- or down-regulate the genes encoding the outer membrane protein synthesis. Beside this, it is able to inhibit the enzymes involved in protection against thermal stress, to affect the synthesis of ATP or to alter citric acid metabolic pathways [434,435].

As mentioned above, carvacrol (**73**) and thymol (**62**) exert a synergic effect, similar to many other combinations of components of essential oils against different common human pathogens (carvacrol/thymol (**73**/**62**), terpinene‑4‑ol/myrcene (**70**/**31**), carvacrol/*p*‑cymene (**73**), eugenol/thymol (**25**/**62**), eugenol/carvacrol (**25**/**73**), cinnamaldehyde/eugenol (**106**/**25**), citronellol/geraniol and others) [436,437,438]. The synergic action of *p*‑cymene and carvacrol (**73**) combination is based on the high affinity of *p*‑cymene to the cytoplasmic membrane and its bonding to the membrane causes its expansion, altering its potential and resulting in its higher sensitivity to the action of carvacrol (**73**) [358]. Some of these combinations of compounds with synergic activities are also present in *Ziziphora* essential oils. The mechanism of thymol (**62**) and carvacrol (**73**) synergism was also elucidated and reviewed [132,439]; however, the mechanistic studies describing the mechanisms of synergy are relatively scarce. Owing to their hydrophobic nature, **73** and **62** interact with the lipid bilayer of cytoplasmic membranes, causing loss of integrity and leakage of cellular material. This effect can, in general, increase the permeability of the membrane to other antimicrobial compounds by general disintegration of the membrane or by formation of a large number of pores.

The synergic activity of some terpenoids can be also observed for other organisms than bacteria, e.g., *Meloidogyne incognita* [440]. Synergic activity was observed also during development of insecticides (pulegone (**53**)/perillaldehyde) [260].

Of note, essential oil components of the thymol (**61**) and carvacrol (**72**) type can act as antagonists, as several essential oils showed lower activity than their single monoterpenic components [281]. The review of Bassolé and Juliani [439] showed some examples of synergic, additive or even antagonistic activity of well-known components of essential oils in different bacterial species.

Carvacrol (**72**) and thymol (**61**) are often mentioned as inhibitors of growth of food-borne pathogens. These pathogens are represented for example by different strains of *Salmonella*, *Shigella*, *E. coli* or *Clostridium*. The activity of the essential oils of *Z. tenuior* and *Z. clinopodioides* against food-borne bacteria has been proven by Aliakbarlu and Shameli [426]. Together with the results of experiments on the antiradical activity of *Z. clinopodioides* essential oil, which showed better activity than *Z. tenuior* [426], *Z. clinopodioides* essential oils can be seen as promising food preservatives. This observation is supported by some other reports that examined the single components identified in *Ziziphora* essential oils (Table 3).

The hypolipidemic activity of aromatic water obtained by mixing the *Z. tenuior* essential oil in water was proven in tests on cholesterol-fed rabbits. However, the levels of measured parameters of hypercholesterolemia were not restored to basal levels [202]. Several patent applications cover the usage of *Ziziphora* in the treatment of some cardiovascular diseases. It is clear that the components of essential oils obtained from *Ziziphora* species can affect the cardiovascular system, as visible from data reviewed in Table 3. The cardiovascular activities of thymol (**61**), carvacrol (**72**), limonene (**38**), α-terpineol (**71**), terpinen4-ol (**70**), linalool (**66**) and menthol (**56**) were reviewed by Santos et al. [221]; however, this review examined a limited number of literature sources. The effects of linalool (**66**) were tested in a human study, and the activity of several compounds (**72**, **38**, **56**, **61**, **70** and **71**) was examined in animal studies. 

Further, linalool (**66**) and 1,8-cineol (**72**) showed hypocholesterolemic activity *via* inhibition of HMG-CoA reductase and inhibition of cholesterol synthesis [318,319]. *Endo*-borneol (**68**) exerts a vasorelaxant effect on rat thoracic aorta artery rings [330] and terpinen-4-ol (**70**) has a relaxant effect on vascular smooth muscle [332].

Several components of *Ziziphora* essential oils were tested and found to possess antioxidant activity, proven in both in vitro and in vivo assays. For example, monoterpenic α-terpinene (**36**), terpinolene (**37**) [217,218], thymol (**62**) [293], borneol (**67**) [324], carvacrol (**73**) [273,294,297,299], and sesquiterpenic caryophylene (**91**) [392] were found to possess antioxidant activity. The antioxidant activity of Lamiaceae essential oils is known, so they can be used in their entirety or their single compounds can be used as food preservatives; moreover, their antibacterial activity and relative non-toxicity makes them more beneficial than some synthetic antioxidants.

#### Potential Toxicity

Monoterpenic pulegone (**53**) is present in many Lamiaceae plants. It is commonly connected with potential toxic effect of so called pennyroyal oil. In high doses, it can cause hepatic failure, central nervous system toxicity, gastritis, renal and pulmonary toxicity, and, in very serious cases, death [451,452]. Assays carried out on mice showed its hepatotoxicity and pulmonary toxicity [451,453]. The toxic potential of pulegone (**53**) is connected to its extensive metabolism in liver, which includes its oxidation to menthofuran, *p*‑cresol and other compounds. These compounds can be further metabolized and cause depletion of glutathione; then, they can covalently bind to proteins and modify their function, causing cell injury [454].

## 3. Conclusions

The traditional medicine of Kazakhstan uses *Ziziphora* species (Lamiaceae) to combat several diseases. Especially, *Z. bungeana* Lam. and *Z. clinopodioides* Lam. are used for the treatment of illnesses connected with cardiovascular system or to combat different infections. We gathered information about four Kazakh *Ziziphora* species, their traditional utilization and the compounds identified in extracts obtained from these plants. This review presented information about each compound and their bioactivities. We can conclude that as a typical example of the Lamiaceae family, phytochemicals present in *Ziziphora* are represented especially by monoterpenic essential oil, phenolic substances belonging to the flavonoids and phenolic acids, and triterpenes. The presence of these particular compounds with confirmed activity can be seen as proof of the traditional use and validation of numerous patent applications. We hope that the review on the compounds isolated from *Ziziphora*, their medicinal uses and published patents will draw the attention of scientists to this very interesting plant with high medicinal potential.

## Figures and Tables

**Table 1:** Phenolic substances isolated from Kazakhstan *Ziziphora* species. (N.f.—not found).

Flavonoids								
Name of Compound and ID	Isolation/Detection in Kazakhstan spp. of *Ziziphora*	Biological Activity	Chemical Structure					
			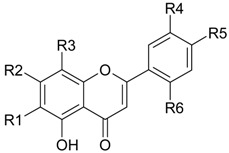					
			R1	R2	R3	R4	R5	R6
Apigenin (**1**)	*Z. tenuior* [38]; *Z. clinopodioides* [4,39,40]	Vasorelaxant activity (IC_50_ 189.4 ± 12.4 µM) [39]; Some inhibitory activity on NO production stimulated by LPS and IFN-γ in RAW 264.7 cells [4] Review on apigenin (**1**) on breast cancer [41] Anticancer activity review [42,43,44] Review on apigenin (**1**) impact on gastric cancer [45] General review on the impact of apigenin (**1**) on health and disease [46]	H	OH	H	H	OH	H
Chrysin (**2**)	*Z. clinopodioides* [39]	Vasorelaxant activity (IC_50_ 347.8 ± 23.9 µM) [39] Neuroprotective activity reviewed [47] Anticancer activity reviewed [48,49] Recent general review on bioactivities of chrysin (**2**) and its derivatives [50]	H	OH	H	H	H	H
Luteolin (**3**)	*Z. clinopodioides* [4]	Some inhibitory activity on NO production stimulated by LPS and IFN-γ in RAW 264.7 cells [4] Anticancer [51,52] Review on anti-inflammatory and neuroprotective effect [53,54] Neurotrophic effects [55] Anti-allergic [56] Anti-atherogenic [57] Cardioprotective [58] General reviews on luteolin (**3**) [59,60]	H	OH	H	OH	OH	H
Thymonin (**4**)	*Z. clinopodioides* [39,61]	Low vasorelaxant activity (IC_50_ not calc.) [39] Toxicity against *Artemia salina* larvae [62] Antiradical [63] Weak antibacterial effect [61]	OH	OCH_3_	OCH_3_	OCH_3_	OH	H
Acacetin (**5**)	*Z. clinopodioides* [39]	Low vasorelaxant activity (IC_50_ not calc.) [39] Inhibition of angiogenesis [64,65] Induction of apoptosis in different cancer cell lines [66,67,68,69,70] Inhibition of TNF-related apoptosis [71] Anticancer [72,73] Induction of melanogenesis in B16F10 cells [74] Cytotoxic against HL-60 cells cells [75] Interaction with telomeres [76] Antimetastatic effect [77,78]	H	OH	H	H	OCH_3_	H
Diosmetin (**6**)	*Z. clinopodioides* [4]	Some inhibitory activity on NO production stimulated by LPS and IFN-γ in RAW 264.7 cells [4] Cytotoxic against HL-60 cells cells [75] Induction of melanogenesis in B16F10 cells [73,74] Review on bioactivity [79]	H	OH	H	OH	OCH_3_	H
Diosmin (**7**)	*Z. clinopodioides* [80]	Antidiabetic activity (reviewed by Abdurrazak et al. [81]) Review on clinical use [82]	H	*O*-Glc-Rha	H	OH	OCH_3_	Hf
Linarin (**8**)	*Z. clinopodioides* [44,80]	Inhibition of mucin production and secretion in airways epithelial cells [83] Hepatoprotective [84] Potential inhibitor of CDK4 in retinoblastoma [85] Neuroprotective [86] Inhibition of acetylcholinesterase [87] Anti-inflammatory [88]; Anti-inflammatory in vivo [89] Depressant effect on CNS [90,91]	H	*O*-Glc-Rha	H	H	OCH_3_	H
Ziziphorin A (**9**)	*Z. tenuior* [39]	n.f.	H	OH	H	H	OCO(CH_2_)_17_CH_3_	H
Ziziphorin B (**10**)	*Z. tenuior* [39]	n.f.	H	OH	H	H	H	OCO(CH_2_)_25_CH_3_)
5,7,2′-trihydroxyflavone-2′-*O*-β-d-glucopyranoside (**11**)	*Z. clinopodioides* [44]	n.f.	H	OH	H	H	H	*O*-Glc
**Other Phenolics**								
**Name of Compound and ID**	**Isolation/Detection in Kazakhstan spp. of *Ziziphora***	**Biological Activity**	**Chemical Structure**					
			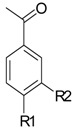					
Acetovanillone (syn. apocynin) (**12**)	*Z. clinopodioides* [39]	Low vasorelaxant activity (IC_50_ not calc.) [39] Review on its potential in treatment of cardiovascular diseases [92] Review on its potential in treatment of neurodegenerative diseases [93]	R1			R2		
			OH			OCH_3_		
4-Hydroxyaceto-phenone (syn. piceol) (**13**)	*Z. clinopodioides* [39]	Low vasorelaxant activity (IC_50_ not calc.) [39]	OH			H		
Acetophenone (**14**)	*Z. tenuior* [94]	Tyrosinase inhibition [95] Acaricidal effect [96]	H			H		
Picein (**15**)	*Z. clinopodioides* ([4,44]	Weak inhibitory activity on NO production stimulated by LPS and IFN-γ in RAW 264.7 cells [4] Glucosidase inhibitor (review by Benalla et al. [97])	*O*-Glc			H		
2-Methoxy-4-vinylphenol (**16**)	*Z. clinopodioides* [15]	n.f.	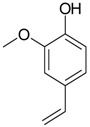					
Caffeic acid (**17**)	*Z. clinopodioides* [80]	Anticancer potential reviewed [98] Protection of endothelial cells (review by Fuentes and Palomo [99]) General review on applications [100]	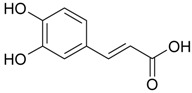					
Ethyl ester of caffeic acid (**18**)	*Z. clinopodioides* [4]	Weak inhibitory activity on NO production stimulated by LPS and IFN-γ in RAW 264.7 cells [4] Antihypertensive [101,102] Anti-inflammatory [103,104,105] Antidiabetic [106] Inhibitory activity against amyloidogenesis [107] Antioxidative [108,109,110,111] Anticancer [112]	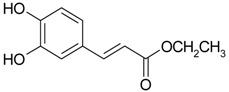					
Rosmarinic acid (**19**)	*Z. clinopodioides* [80]	Review on pharmaceutical and clinical usage [113] Neuroprotective—review [114] General review on applications [115] Review on anticancer potential [116]	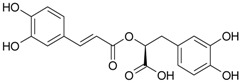					
Salicylic acid (**20**)	*Z. clinopodioides* [80]	Effect on cardiovascular system reviewed [117] Pharmacological importance reviewed [118]	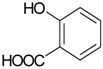					
Benzoic acid (**21**)	*Z. clinopodioides* [4]	Weak inhibitory activity on NO production stimulated by LPS and IFN-γ in RAW 264.7 cells [4] Properties reviewed here [119]	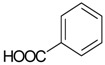					
(*Z*)-3-Hexen-1-ol benzoate (**22**)	*Z. tenuior* [94]	n.f.	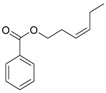					
Benzylalcohol glucoside (**23**)	*Z. clinopodioides* [4]	Weak inhibitory activity on NO production stimulated by LPS and IFN-γ in RAW 264.7 cells [4] Antihypertensive effect [120] Low anti-inflammatory activity [121] Neuroprotective effect [122] Weak inhibition of TPA-induced EBV-EA activation [123]	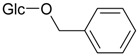					
Phenethylalcohol glucoside (**24**)	*Z. clinopodioides* [4]	Weak inhibitory activity on NO production stimulated by LPS and IFN-γ in RAW 264.7 cells [4] Neuroprotective effect [122] Antiradical activity, weak ACE-inhibitory activity [124] Inhibition of osteoclast differentiation [125]	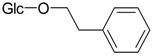					
Eugenol (**25**)	*Z. tenuior* [94]	Insecticidal (against *L. serricorne*) [126] Acaricidal activity [127] Antibacterial [128] Inhibition of tyrosine kinase [129] Review on possible antidepressive activity [130] Review on antibacterial effect against cariogenic bacteria [131] Review on possible synergy of eugenol containing essential oils and eugenol (25) with antibiotics [132] Review on antioxidative effect [133] General properties reviewed here [134]	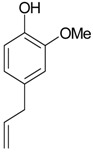					

**Table 2:** Triterpenic substances isolated from Kazakhstan *Ziziphora* species. (N.f.—not found).

Triterpenes and Sterols			
Name of Compound and ID	Isolation/Detection in Kazakhstan spp. of *Ziziphora*	Biological Activity	Chemical Structure
Oleanolic acid (**26**)	*Z. clinopodioides* [4,80,143]	Weak inhibitory activity on NO production stimulated by LPS and IFN-γ in RAW 264.7 cells [4] Review on effect on vascular functions [140] General review on bioactivity and mechanisms of effect [143,144]	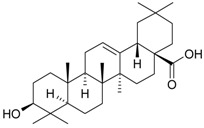
Ursolic acid (**27**)	*Z. clinopodioides* [4,80,143]	Weak inhibitory activity on NO production stimulated by LPS and IFN-γ in RAW 264.7 cells [4] Cytotoxicity against HL-60 and LLC cell line [145] Recent general review on bioactivity [142] General review on bioactivity [146] Review on anticancer potential [147,148]	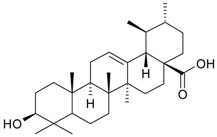
Maslinic acid (**28**)	*Z. clinopodioides* [4]	Weak inhibitory activity on NO production stimulated by LPS and IFN-γ in RAW 264.7 cells [4] Cytotoxicity against HL-60 and LLC cell line [145] Recent review on bioactivity [139,144] Review on anti-inflammatory potential [149]	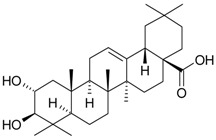
Daucosterol (**29**)	*Z. clinopodioides* [44]	Immunoregulatory effect [150] Anti-inflammatory activity [151]; anti-inflammatory in ear edema assay [152]; topical anti-inflammatory activity in the mouse ear edema model [153]; weak 5-LOX inhibitory activity [154] DPPH and ABTS; scavenging effect [155]; antioxidant [156]; inhibitory effect on nitric oxide production in LPS-activated RAW264.7 cells [157] Antinociceptive [158] Some anticomplementary activity [159] Neuroprotective [160,161]; promotion of proliferation of neural stem cells [162]; Inhibition of acetylcholinesterase [163] Inhibition of cancer cell proliferation [164], induction of apoptosis [165], antiproliferative [166], cytotoxic [167]; antiproliferative activity against HL-60, K562, HepG2 and CNE-1 cell lines [168] inhibition of MDA-MB-231 cancer cell migration [169]; Ability to activate PPARγ and PPARβ [170] Inhibition of α-glucosidase [171] Antibacterial effect against *E. coli* [172], against *H. pylori* and *S. aureus* [173]; against *E. coli* and *S. aureus* [174]; against *Bacillus subtilis* and *S. aureus* [175] Inhibitory effect on reverse transcriptase [176] Suppression of HCl/ethanol-induced gastric lesions [177] Inhibitory activity against osteoclast differentiation by suppressing TRAP activity in RANKL-induced RAW 264.7 macrophage cells [178]	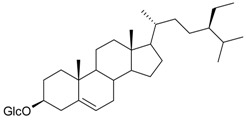

**Table 3:** Mono and sesquiterpenic substances isolated from Kazakhstan *Ziziphora* species (N.f.—not found).

Monoterpenes			
Name of Compound and ID	Isolation/Detection in Kazakhstan spp. of *Ziziphora* (Percentage Content when Given)	Biological Activity	Chemical Structure
Myrcene (syn. β-myrcene) (**30**)	*Z. clinopodioides* (0.6%–1.9%) [9,15,185]	Antibacterial, antitermitic, antifungal [187] Antioxidant (DPPH) [188] Some cytotoxic activity [189] Low fumigant activity against various species of insects [190,191] Sedative and motor relaxant effects in mice [192] Anti-inflammatory activity in the mouse model of pleurisy induced by LPS [193] Protective effect against *t*‑BOOH induced mutagenesis [194] Immunomodulatory and protective effects against the immunotoxicity induced by TCDD in rats [195]; antioxidant activity against TCDD‑induced oxidative stress in rats liver [196]; neuroprotective after global cerebral ischemia/reperfusion-mediated oxidative and neuronal damage in mouse [197] Gastroprotective effect against various ulcerogenic agents [198] Analgesic activity reviewed [199] Review on metabolism and toxicity [200]	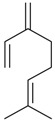
	*Z. clinopodioides* subsp. *rigida* (0.2%) [21]		
	*Z. clinopodioides* subsp. *bungeana* (0.3%) [201]		
	*Z. tenuior* (0.1%) [179,202]		
*(Z)-*β*-*Ocimene (**31**)	*Z. clinopodioides* (1.1%) [184]	Antibacterial [187] Antifungal (*Candida* strains, *Cryptococcus neoformans*, *Epidermophyton floccosum*, *Microsporum canis* and *M. gypseum*, *Trychophyton mentagrophytes* and *T. verrucosum*, *Aspergillus flavus*, *A fumigatus*, *A. niger*) [203,204]	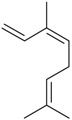
	*Z. clinopodioides* subsp. *bungeana* [201]		
*(E)-*β-Ocimene (**32**)	*Z. clinopodioides* (1.2%) [184]	Antifungal (*Candida* strains, *Cryptococcus neoformans*, *Epidermophyton floccosum*, *Microsporum canis* and *M. gypseum*, *Trychophyton mentagrophytes* and *T. verrucosum*, *Aspergillus flavus*, *A fumigatus*, *A. niger*) [203,204]	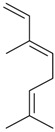
3,7-Dimethyl-1,3,7-octa-triene (**33**)	*Z. tenuior* [94]	n.f.	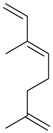
α-Thujene (**34**)	*Z. clinopodioides* (0.1%–1.2%) [15,184]	n.f.	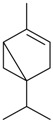
	*Z. clinopodioides* subsp. r*igida* (0.1%) [21]		
	*Z. clinopodioides* subsp. *bungeana* [201]		
	*Z. tenuior* (0.48%) [94,179]		
α-Phellandrene (**35**)	*Z. clinopodioides* (0.3%) [184]	Insecticidal activity against various species of insects [192,193]; larvicidal activity against various mosquito species [205,206] Antinociceptive activity assessed in various chemical‑induced nociception models in rodents [207,208] Antidepressant activity in rats [208] Induction of autophagy in human liver tumour cells [209]; induction of necrosis [210]; induction of apoptosis in mice leukaemia WEHI‑3 cells in vitro [211]; promotion of the immune response by increasing the level of T‑cells, monocytes and macrophages in BALB/c mice in vivo [212]	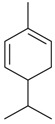
α-Terpinene (**36**)	*Z. clinopodioides* (2.0%) [184]	Inhibition of P-glycoprotein-mediated transport of different substances [213] Suppression of CNS in mice [214,215] Some topical anti-inflammatory activity in carrageenan‑induced paw edema in rats [216] Antioxidant activity in various free radical scavenging tests [217,218] Antifungal activity tested against some food spoilage yeasts [219] Larvicidal activity against mosquitoes *Aedes aegypti* and *A. albopictus* [206] Antiviral activity against herpes simplex virus type 1 in vitro [220] Review on some effects on cardiovascular system [221] Trypanocidal activity against *Trypanosoma evansi* [222]	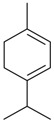
	*Z. clinopodioides* subsp. *rigida* [21]		
	*Z. clinopodioides* subsp. *bungeana* (0.1%) [201]		
Terpinolene (**37**)	*Z. clinopodioides* (0.1%–0.2 %) [184]	Inhibition of P-glycoprotein-mediated transport of different substances [213] Anti-inflammatory (inhibition of NO production in LPS-stimulated RAW-264.7 macrophages) [223] Inhibition of acetylcholinesterase and butyrylcholinesterase [224] Suppression of CNS in mice [214,215,225] Antiproliferative (tested on primary rat neurons and N2a neuroblastoma cells) [226]; antiproliferative effect on K562 cells [227]; some cytotoxic activity [190]	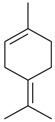
	*Z. clinopodioides* subsp. r*igida* (0.5%) [21]		
	*Z. clinopodioides* subsp. *bungeana* (0.1%) [201]		
	*Z. tenuior* (0.19%) [94,179]		
		Insecticidal activity against *Callosobruchus chinensis* and *Sitophilus oryzae* [228]; larvicidal activity tested against various mosquito species [205,206,229] Antioxidant activity in various free radical scavenging tests [217,218]; protective effect against LDL‑oxidation [230] Review on some effects on cardiovascular system [221] Antiviral activity against influenza A⁄PR⁄8 virus subtype H1N1 [231]	
Limonene (**38**)	*Z. tenuior* (0.51%–7.82%) [232]	Insecticidal (against red imported fire ant) [233] Fumigation activity against stored-product pest insects [234] Anxiolytic effect in mice [225] Gastroprotective in rats [235] Pharmacological importance and properties reviewed [190,236,237] Analgesic activity reviewed [199] Review on some effects on cardiovascular system [221] Review on metabolism and toxicity [200]	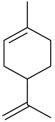
	*Z. clinopodioides* [11,15]		
*p*-Mentha-1(7,8)-diene (syn. pseudolimonene) (**39**)	*Z. tenuior* (0.04%) [179]	n.f.	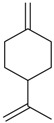
*p*-Mentha-3,8-diene (**40**)	*Z. tenuior* (1.65%) [179]	n.f.	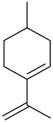
	*Z. clinopodioides* subsp. *bungeana* [201]		
4-Methyl-1-(1-methyl-ethenyl)-cyclohexene (syn. 3,8-*p*-menthadiene) (**41**)	*Z. tenuior* [94]	n.f.	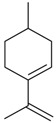
3-Methyl-6-(1-methyl-ethylidene)-cyclohexene (syn. Isoterpinolene, 2,4-*p*-menthadiene) (**42**)	*Z. tenuior* [94]	n.f.	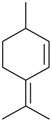
3-Isopropenyl-5,5-di-methyl-cyclopentene (**43**)	*Z. tenuior* [94]	n.f.	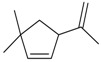
Menthone (**44**)	*Z. clinopodioides* (6.2%–13.3%) [238]	Antifungal effect against *C. albicans*, synergy with fluconazol [239]; antimicrobial activity against tested strains of bacteria, yeast and pathogenic fungi [240,241] Insecticidal activity tested against various stored grain pests and vectors [242]; moderate insecticidal activity against *Sitophilus zeamais* [243] Antidepressant-like effects in an unpredictable chronic mild stress mouse model of depression [244]	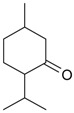
Piperitone (**45**)	*Z. clinopodioides* (4.18%) [11]	Repellent activity against ants of the genus *Crematogaster* [245]; insecticidal activity against larvae of *Spodoptera littoralis* [246] and against *Callosobruchus maculatus* [247] Increase in antimicrobial activity of furazolidone and nitrofurantoin against bacteria of the family Enterobacteriaceae [248,249] Fungicidal activity against *Aspergillus flavus* [250]	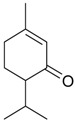
	*Z. clinopodioides* subsp. *rigida* (1.4%) [21]		
	*Z. clinopodioides* subsp. *bungeana* (0.6%) [201]		
Piperitenone (**46**)	*Z. clinopodioides* (5.3%) [11]	Antibacterial activity against 52 Gram-positive and Gram-negative bacterial species, disc diffusion method [11] Insecticidal activity against *Sitophilus zeamais* [243]	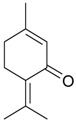
	*Z. clinopodioides* subsp. *rigida* (17.4%) [21]		
*p-*Menth-4-en-3-one (**47**)	*Z. tenuior* (0.5%) [202]	n.f.	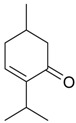
Piperitone oxide (**48**)	*Z. persica* (0.32%) [251]	Antibacterial activity against 19 Gram-positive and Gram-negative bacterial species [252]	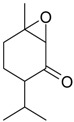
Piperitenone oxide (**49**)	*Z. clinopodioides* (0.16%) [11]	Antimicrobial activity against strains of bacteria, yeast and pathogenic fungi [241,252] Insecticidal activity against the West Nile virus mosquito *Culex pipiens* larvae [253] and against various stages of *Anopheles stephensi* [254] Antinociceptive activity in acetic acid‑induced writhing test and in the second phase of formalin test [255] Antiviral activity against herpes simplex virus type 1 [256]	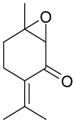
Verbenone (**50**)	*Z. clinopodioides* subsp. *rigida* (0.2%) [21]	Antifungal activity against *Botrytis cinerea* [257] Fumigation activity against stored-product pest insects [234] Insecticidal activity against *Acanthoscelides obtectus* [258]	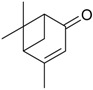
2-Acetyl-4,4-dimethyl-cyclopent-2-enone (**51**)	*Z. tenuior* (2.49%) [94]	n.f.	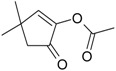
2-Isopropyl-5-methyl-3-cyclohexen-1-one (**52**)	*Z. tenuior* [94]*,* *Z. tenuior* (1.6%) [179]	n.f.	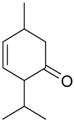
Pulegone (**53**)	*Z. tenuior* (86.29%–87.06%) [259]	Insecticidal [260] Fumigation activity against stored-product pest insects [234] Anti-inflammatory activity reviewed [261] Analgesic activity reviewed [199] Review on metabolism and toxicity [200]	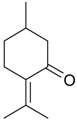
	*Z. clinopodioides* (45.8%) [21]		
3-Methyl-6-(1-methyl-ethenyl)-2-cyclohexen-1-one (syn. Isopiperitenone) (**54**)	*Z. tenuior* (0.3%–1%) [94,259]	n.f.	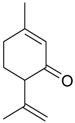
Carvotanacetone (**55**)	*Z. tenuior* [94]	Cytotoxic activity against MCF‑7 and Hep‑G2 cells [262] Antifungal activity against tested phytopathogenic fungi [263]	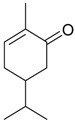
Menthol (**56**)	*Z. clinopodioides* (9.13%) [11]	Antifungal effect against *Candida albicans*, synergy with fluconazol [239] Fumigant activity [264] Analgesic, antifungal, antibacterial, antipruritic, anticancer, anti-inflammatory, antitussive, antiviral, insecticidal activity reviewed in Kamatou et al. [265] Gastroprotective effect in gastric ulcers induced by ethanol or indomethacin in Wistar male rats and also antidiarrheal and antiperistaltic effect; anti-apoptotic, antioxidant and anti-inflammatory activities [266,267] Allosteric modulation of human α3β4 nicotinic acetylcholine receptors [268] Analgesic activity reviewed [199] Review on some effects on cardiovascular system [221] Review on metabolism and toxicity [200]	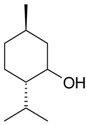
	*Z. clinopodioides* subsp. *rigida* (0.1%) [21]		
Menthofuran (**57**)	*Z. tenuior* (0.1%) [182]	Acetyl and butyrylcholinesterase inhibitory activity [269]	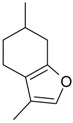
Neomenthol (**58**)	*Z. clinopodioides* subsp. *rigida* (2.1%) [21]	Antibacterial activity against *Escherichia coli* [270] Acaricidal activity against *Tyrophagus putrescentiae* [271] Sedative in the pentobarbital‑induced sleep test in mice [272]	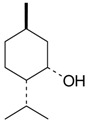
	*Z. clinopodioides* subsp. *bungeana* [201]		
*Neo-iso*-menthol (**59**)	*Z. clinopodioides* subsp. *bungeana* (0.3%) [201]	n.f.	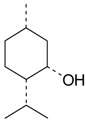
	*Z. clinopodioides* (0.12%–0.25%) [15]		
Menthyl acetate (**60**)	*Z. clinopodioides* (0.1%) [184]	n.f.	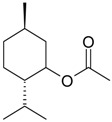
Isomenthyl acetate (**61**)	*Z. clinopodioides* subsp. *rigida* (0.5%) [21]	n.f.	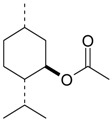
Thymol (**62**)	*Z. clinopodioides* (0.17%–53.6%) [15,184]	Antibacterial (*Bacillus cereus*, *Micrococcus flavus*, *S. aureus*, *Listeria monocytogenes*, *E. coli*, *P. aeruginosa*, *Proteus mirabilis*, *Salmonella typhimurium*) [261,273,274] Antifungal (*Trichophyton* *mentagrophytes*, *T. interdigitale*, *T. rubrum*, *T. erinaceum*, *T. soudanense*, *T. violaceum*, *Microsporum canis*, *M. gypseum*, *Epidermophyton flocosum*, *A. fumigatus*, *Scopulariopsis brevicaulis*, *Scytalidium dimidiatum*, *C. albicans*, *Cryptococcus neoformans*, *Malassezia pachydermatis* [273]); against *Botrytis cinerea* [275]; against *Penicillium funiculosum* and *P. ochrochloron*, *Aspergillus fumigatus*, *A. niger*, *A. flavus*, *A. ochraceus*, *C. albicans*, *Trichoderma viride*) [187]; antifungal (*Candida albicans*, *C. tropicalis*, *Saccharomyces cerevisiae*, *Cryptococcus neoformans*, *Microsporum gypseum*, *Trichophyton rubrum*, *T. mentagrophytes*), antimicrobial (*E. coli*, *P. aeruginosa*, *Yersinia enterocolitica*, *Salmonella enteritidis*) activity [276]; antifungal (yeasts, dermatophyte and *Aspergillus* strains) [277] Antifungal activity against various *Candida* strains and synergy with fluconazole [278] Review on possible synergy of 62 containing essential oils and thymol (**62**) with antibiotics [132] Antileshmanial (against *L. chagasi*) [279] Antimalarial (*P. falciparum*) [280] Low antinematocidal activity [281] Antiviral (HSV-1) [282] Cytotoxic (HeLa, B16, MCF-7, 3T3, MRC-5 cells) [283]; against P815 and PBMC [284]; induction of apoptosis [285]; cytotoxic effects on acute promyelotic cancer cell line HL‑60 [286]	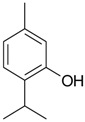
	*Z. clinopodioides* subsp. *rigida* (8%) [21]		
	*Z. tenuior* [94]		
		Anti-genotoxic (bleomycin-induced DNA damage) [287] Anti-inflammatory activity in carrageenan-induced paw edema, MPO activity and peritonitis in rats [288]; anti-inflammatory activity in LPS‑stimulated mouse mammary epithelial cells *via* inhibition of the NF‑κB and MAPKs signalling pathways [289]; anti-inflammatory effect in ovoalbumin‑induced mouse asthma, possibly through inhibiting NF‑κB activation [290]; inhibition of *S. aureus* internalization into bovine mammary epithelial cells by inhibiting NF-κB activation [291] Review on anti-inflammatory, antioxidant, and immunological effects [292] Protective effect on radiation‑induced apoptosis in Chinese hamster lung fibroblast V79 cells [293]; DNA‑protective effects against H_2_O_2_-induced DNA lesions in human hepatoma HepG2 cells [294]; hepatoprotective effect against *t*‑BHP‑induced oxidative damage in Chang liver cells [84]; protective effect against cisplatin‑induced nephrotoxicity in rats [295]; protective effect against UVA‑ and UVB‑induced lipid peroxidation in NCTC 2544 cell line [296]; antioxidant [273,297,298,299] Review on some effects on cardiovascular system [221] Gastroprotective effects in the acute and chronic ulcer models in rats [300] Positive allosteric modulator of the GABA_A_ receptors in primary cultures of mouse cortical neurons [301] Anti-hyperglycemic and anti-hyperlipidemic activity in high fat‑induced type 2 diabetic C57BL/6J mice [302] and protective effect in nephropathy [303] Analgesic activity reviewed [199] Review on metabolism and toxicity [200]; general review on thyme and **62** [304]	
*cis-*Sabinene hydrate (**63**)	*Z. clinopodioides* (0.3%–2.2%) [184]	Antiviral (HSV-1) [282]	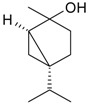
	*Z. clinopodioides* subsp. *bungeana* [201]		
*trans-*Sabinene hydrate (**64**)	*Z. clinopodioides* subsp. *rigida* (0.1%) [21]	Antiviral (HSV-1) [282] Moderate repellent (against tick *Amblyomma americanum*) [305]	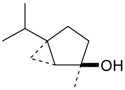
	*Z. tenuior* [94]		
*p*-Menth-3-en-8-ol (**65**)	*Z. clinopodioides* (2.7%) [185]	n.f.	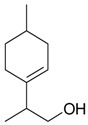
	*Z. clinopodioides* subsp. *rigida* (12.5%) [21]		
	*Z. tenuior* (53.97%) [179]		
Linalool (**66**)	*Z. clinopodioides* (1.8%–7.9%) [184]	Antibacterial [128,187,306] Antimycotic (*Candida albicans*) [307], against *M. ramamnianus* [308] Repellent against mosquitoes [309] Molluscicidal (against snail *Oncomelania hupensis*) and cercarcicidal (against *Schistosoma japonicum*) [310] Anti-inflammatory (in carrageenan‑induced edema and inhibition of hyperalgesia induced by L‑glutamate and prostaglandin E_2_ in rats) [311]; anti-inflammatory (through inhibition of the expression of TNF-α and IL-6 in LPS-stimulated RAW 264.7 cells) [312]; anti-inflammatory (in cigarette smoke-induced ALI in mice through inhibiting NF‑κB activation) [313] Cytotoxic (human amelanotic melanoma cell line C32, renal cell adenocarcinoma ACHN, hormone-dependent prostate carcinoma LNCaP) [314]; induction of apoptosis (variety of human leukaemia cells) [315,316,317] Hypocholesterolemic (through inhibition of HMG-CoA reductase and conversion of lanosterol to cholesterol) [318,319] Protective effect against *t*‑BOOH induced mutagenesis [194] Review on some effects on cardiovascular system [221] Sedative and anxiolytic [320]; antidepressant [321]; GABA_A_ receptor modulation [322] Analgesic activity reviewed [199] Review on metabolism and toxicity [200]	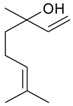
	*Z. clinopodioides* subsp. *rigida* [21]		
Borneol (**67**)	*Z. clinopodioides* (0.9%–1.2%) [184]	Inhibition of nicotinic acetylcholine receptor [323] DNA-protective effects against H_2_O_2_ in primary rat hepatocytes and testicular cells [324] Anti-inflammatory in an ALI model in mice through inhibition of the NF-κB and MAPKs signalling pathways [325]; suppression of expression of IL‑1β and IL‑6 in TNBS‑induced colitis in mice [326] Penetration enhancer [327]; increasing of the brain bioavailability of different drugs [328] Antibacterial (multi-drug resistant *E. coli*) [329] Antiviral (HSV-1) [282]	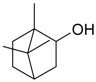
*endo*-Borneol (**68**)	*Z. tenuior* (0.14%) [202]	Vasorelaxant effect on rat thoracic aorta artery rings [330] Positive modulation of the activation of GABA_A_ receptors [331] Potentiation of SeC-induced apoptosis in human hepatocellular carcinoma cells [332] Prolonging anaesthesia time of propofol by inhibiting its glucuronidation [333]	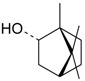
Bornyl acetate (**69**)	*Z. clinopodioides* (3.3%) [184]	Antifungal (against *Pyrenophora avenae*) [334] Insecticidal (against *Callosobruchus chinensis* and *Sitophilus oryzae*) [228] Anti-inflammatory in an ALI model in mice [335] and in human chondrocytes [336] Antiabortive in pregnant mice [337] Cytotoxic ((Eca-109, HepG2, HT29, MDA-MB-231, PC-3, SGC7901, SW1990 and U2-OS) and a normal cell line (HL-7702) [338]	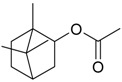
Terpinen-4-ol (**70**)	*Z. clinopodioides* (0.36%–18.2%) [15,184]	Sedative and anaesthetic (on silver catfish juveniles) [339]; depressant effect on the CNS and significant anticonvulsant activity probably due to interaction with GABA receptors [340,341] Low inhibition of acetylcholinesterase and butyrylcholinesterase [224] Relaxant effect on vascular smooth muscle [342] Review on some effects on cardiovascular system [221] Anticancer (melanoma) [343]; antiproliferative activity in two murine cancer cell lines through induction of necrosis and cell cycle arrest [344]; induction of apoptosis in human non-small cell lung cancer [345]; anti-tumoral activity in human melanoma cells by induction of caspase-dependent form of apoptosis [346] Antiviral activity against influenza A⁄PR⁄8 virus subtype H1N1 [231] Anti-inflammatory (inhibition of NO production in LPS-stimulated RAW-264.7 macrophages) [223]; anti-inflammatory [216]; anti-inflammatory activity in a murine model of oral candidiasis [347]; suppression of the production of TNFα, IL‑1β, IL‑8, IL‑10 and PGE_2_ by LPS‑activated monocytes [348] Antibacterial against MRSA and CoNS [349] Low antinematocidal activity [281] Antimycotic (*C. albicans*) [307,350] Insecticidal (against *L. serricorne*) [351] Trypanocidal activity against *Trypanosoma evansi* [222]	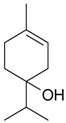
	*Z. clinopodioides* subsp. *rigida* (0.4%) [21]		
	*Z. clinopodioides* subsp. *bungeana* (0.6%) [201]		
	*Z. tenuior* (0.08%) [202]		
α-Terpineol (**71**)	*Z. clinopodioides* (0.3%–5.3%) [184]	Antifungal [187,350,352], antimicrobial activity [306] Antiviral activity against influenza A⁄PR⁄8 virus subtype H1N1 [231] Enhancement of GABA modulation [353] Anticancer (melanoma cells) [343] Insecticidal (against *L. serricorne*) [351] Inhibition of the acetic acid-induced writhing and formalin-induced nociception in mice [354] Analgesic activity reviewed [199] Review on metabolism and toxicity [200]	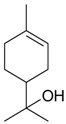
1,8-Cineol (**72**)	*Z. clinopodioides* (5.4%–21.6%) [185]	Antimicrobial activity (MDR resistant bacteria) [355] Hypocholesterolemic (through inhibition of HMG‑CoA reductase and conversion of squalene to lanosterol [319] Analgesic activity reviewed [199] Review on some effects on cardiovascular system [221] Review on metabolism and toxicity [200]	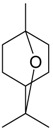
Carvacrol (**73**)	*Z. clinopodioides* (8.7%) [184]; *Z. clinopodioides* (52.7%) [183]	Antifungal (*Penicillium funiculosum* and *P. ochrochloron*, *Aspergillus fumigatus*, *A. niger*, *A. flavus*, *A. ochraceus*, *C. albicans*, *T. viride*) [187,273,277] Antibacterial (*B. cereus*, *M. flavus*, *S. aureus*, *L. monocytogenes*, *E. coli*, *P. aeruginosa*, *P. mirabilis*, *S. typhimurium*) [273,274]; against *B. cinerea* [275]; active against food spoilage microorganisms [356,357,358] Low antinematocidal activity [281] Antileishmanial (against *L. chagasi*) [279] Antiviral (HSV-1) [283]; antiviral activity on enteric viruses [359] Review on synergic effect with antibiotics [132] Cytotoxic (HeLa, B16, MCF-7, 3T3, MRC-5 cells) [284]; against P815 and PBMC [284]; antiproliferative effects on a human metastatic breast cancer cell line, MDA‑MB 231 [360]; induction of apoptosis in HL‑60 and Jurkat cells by mitochondria‑mediated pathway through the involvement of caspase‑3 [361] Cytotoxic effect on the intestinal cell line Caco‑2 [362]; inhibition of growth of *N‑ras* oncogene transformed mouse myoblast cells [363] DNA‑protective effects against H_2_O_2_-induced DNA lesions in human hepatoma HepG2 cells [295]; antioxidant [273,297,299] Anti-inflammatory (inhibition of NO production in LPS-stimulated RAW-264.7 macrophages) [223]; anti-inflammatory effect by reducing the production of IL‑1β and prostanoids, possibly through the induction of IL‑10 release [364] Anti-genotoxic [287] Anxiolytic, GABA_A_ receptor modulation [322,365] Bronchodilatory effect in guinea pigs [366] Antinociceptive activity in mice in the acetic acid-induced abdominal constriction, formalin and hot-plate tests [367] Acetyl‑ and butyrylcholinesterase inhibitory activity [269,368] Analgesic activity reviewed [199] Review on anti-inflammatory, antioxidant, and immunological effects [292] Review on some effects on cardiovascular system [221] General reviews on **73** [369,370]	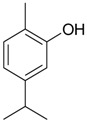
	*Z. tenuior* [94]		
2-Methyl-5-(1-methyl-ethyl)phenol acetate (syn. carvacryl acetate) (**74**)	*Z. tenuior* [94]	Anthelmintic *Haemonchus contortus* [371] Anti-inflammatory effect through inhibition of edema induced by carrageenan, histamine, serotonin or PGE_2_ [372] Anxiolytic-like effect probably through acting on the GABAergic system [373,374]	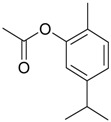
*trans*-*p*-Mentha-2,8-di-enol (syn. *trans*-Isopiperitenol) (**75**)	*Z. tenuior* [94]	n.f.	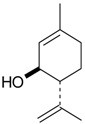
*cis-*Verbenol (**76**)	*Z. clinopodioides* subsp. *rigida* (0.1%) [21]	Antibacterial (multi-drug resistant *E. coli*) [329] Insecticidal activity (against *Lasioderma serricorne*) [351] GABA_A_ receptor modulation [322] Repellent activity against *Anopheles gambiae* [375] Anti-ischemic and anti-inflammatory activity [376]	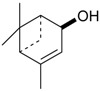
*trans-*Verbenol (**77**)	*Z. clinopodioides* subsp. *rigida* (0.1%) [21]	Antibacterial (multi-drug resistant *E. coli*) [329] GABA_A_ receptor modulation [322]	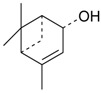
Cuminyl aldehyde (syn. cumaldehyde) (**78**)	*Z. clinopodioides* subsp. *rigida* (0.8%) [21]	Tyrosinase inhibitory activity [377] Suppression of melanin formation in cultured murine B16‑F10 melanoma cells [378]	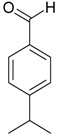
	*Z. clinopodioides* (0.12%–0.24%) [15]		
Ziziphoroside A (**79**)	*Z. clinopodioides* [4]	Weak inhibitory activity on NO production stimulated by LPS and IFN-γ in RAW 264.7 cells [4]	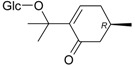
Ziziphoroside B (**80**)	*Z. clinopodioides* [4]	Weak inhibitory activity on NO production stimulated by LPS and IFN-γ in RAW 264.7 cells [4]	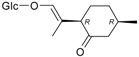
Ziziphoroside C (**81**)	*Z. clinopodioides* [4]	Weak inhibitory activity on NO production stimulated by LPS and IFN-γ in RAW 264.7 cells [4]	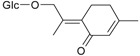
Schizonepetoside C (**82**)	*Z. clinopodioides* [4]	Weak inhibitory activity on NO production stimulated by LPS and IFN-γ in RAW 264.7 cells [4]	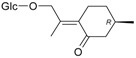
Schizonepetoside A (**83**)	*Z. clinopodioides* [4]	Some inhibitory activity on NO production stimulated by LPS and IFN-γ in RAW 264.7 cells [4]	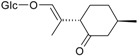
9-*O*-Glucopyranosyl-*p*-menthan-3-one (**84**)	*Z. clinopodioides* [4]	Weak inhibitory activity on NO production stimulated by LPS and IFN-γ in RAW 264.7 cells [4]	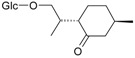
4aα,7α,7aα-Nepeta-lactone (**85**)	*Z. tenuior* (0.5%) [94,179]	Some activity against *Helicobacter pylori* [379] and repellent activity against mosquitoes [380]	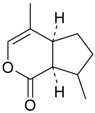
**Sesquiterpenes**			
**Name of Compound and ID**	**Isolation/Detection in Kazakhstan spp. of *Ziziphora* (Percentage Content When Given)**	**Biological Activity**	**Chemical Structure**
Germacrene B (**86**)	*Z. clinopodioides* (1.1%) [11]	n.f.	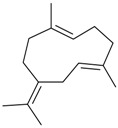
Germacrene D (**87**)	*Z. clinopodioides* (0.24%–4.0 %) [15,184]	Effective aphid repellent [381]	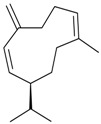
	*Z. clinopodioides* subsp. *rigida* (1.1%) [21]		
	*Z. tenuior* (0.13%) [94,179]		
	*Z. clinopodioides* subsp. *bungeana* (0.3%) [201]		
Bicyclogermacrene (**88**)	*Z. clinopodioides* (0.6%) [185]	Low antibacterial effect (*S. aureus*, *B. cereus, A. baumanii*, *E. coli*, *P. aeruginosa*) [382]	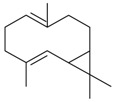
	*Z. clinopodioides* subsp. *bungeana* (0.1%) [201]		
β-Bisabolene (**89**)	*Z. clinopodioides* (0.2%) [184]	Synergistic antibacterial activity with ampicillin against strain of *S. aureus* [383]	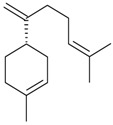
	*Z. tenuior* [94]		
Caryophyllene oxide (β-caryophyllene oxide) (**90**)	*Z. clinopodioides* (0.5%) [184]	Antitermitic [187], antifungal [308,384], antimicrobial [385] Analgesic and anti‑inflammatory activity in tested mice [386] Modest cytotoxic activity against tested human tumor cell lines [387] Anti-cancer effects through the modulation of the PI3K/AKT/mTOR/S6K1 and MAPK signalling [388]	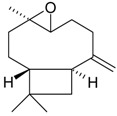
	*Z. tenuior* (0.11%–0.32%) [94,179,202]		
Caryophyllene (syn. β-caryophyllene, *trans*-caryophyllene, (*E*)-caryophyllene) (**91**)	*Z. tenuior* (0.22%) [94,179]	Antifungal, antibacterial [187] Antiprotozoal (*T. cruzi*, *L. infantum*) [389] Fumigant against *Lasioderma serricorne* [390] Antihyperglycemic effect by decreasing blood glucose and increasing plasma insulin in diabetic rats [391] Antioxidant effect and inhibition of 5‑lipoxygenase in CCl_4_‑induced fibrosis in rats [392] Anti-inflammatory effect through the inhibition of TNFα and PGE_2_ and it is also effective in reducing PAF‑, bradykinin‑, ovoalbumin‑induced mouse paw edema [393,394] Low inhibition of acetylcholinesterase and butyrylcholinesterase [224] Review on metabolism and toxicity [200]	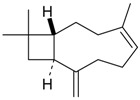
α-Humulene (syn. α-Caryophyllene) (**92**)	*Z. clinopodioides* (2.7%–4.5%) [184]	Antibacterial activity against *Propionibacterium acnes* (MIC of 3.13 μg/mL) [395] Antiproliferative activity against several cancer cell lines (MCF-7, PC-3, M4BEU, CT-26, human amelanotic melanoma cell line C32, renal cell adenocarcinoma ACHN, hormone-dependent prostate carcinoma LNCaP, A-549 and human colon adenocarcinoma cell line DLD-1) [396,397,398,399,400,401] Anti-inflammatory effect through the inhibition of TNFα, IL‑1β and PGE_2_ and it is also effective in reducing PAF‑, bradykinin‑, ovoalbumin‑ and histamine‑induced mouse paw edema [393,394]	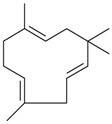
δ-Cadinene (**93**)	*Z. tenuior* [94]	Antimicrobial activity against *Streptococcus pneumoniae* strains resistant to β-lactamic antimicrobials (MIC of 31.25 μg/mL) [402] Antibacterial activity against *Propionibacterium acnes* (MIC of 3.13 μg/mL) [395] Antileishmanial effect against *L. donovani* [403]	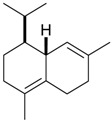
*τ-*Cadinol (**94**)	*Z. tenuior* [94]	Weak antimite activity against *Dermatophagoides pteronyssinus* [404] Good antifungal activity against brown rot fungi *Laetiporus sulphureus* and weak antifungal activity against white rot fungi *Coriolus versicolor* [405] Anti-wood-decay fungal activity [274,406]	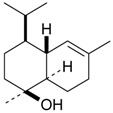
Patchouli alcohol (**95**)	*Z. clinopodioides* (0.1%–1.04 %) [15]	Anti-inflammatory activity through inhibition of over-expression of iNOS and IL-6 in LPS-stimulated RAW264.7 and TNF-α HT-29 cells [407,408]; anti-inflammatory effect in vivo in rats [409] Gastroprotective [410] Review on metabolism and toxicity [200]	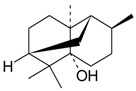
(*Z*)-6,10-dimethyl-5,9-undecadien-2-one (syn. *Z*-geranylacetone, nerylacetone) (**96**)	*Z. tenuior* [94]	n.f.	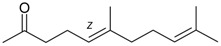
Cyclohexane, 1-ethenyl-1-methyl-2-(1-methylethenyl)-4-(1-methylethylidene) (**97**)	*Z. tenuior* [94]	n.f.	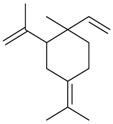
Spathulenol (**98**)	*Z. tenuior* [94]	Antimicrobial activity against *S. aureus* and *P. mirabilis* [385]	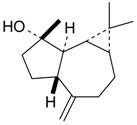
Bicyclo[5.2.0]no-nane, 2-methylene-4,8,8-tri-methyl-4-vinyl (**99**)	*Z. tenuior* [94]	Antimicrobial, anti-inflammatory, antihyperlipidemic, antioxidant activities [411] Repellent activity against *Tribolium castaneum* and *Myzus persicae* [412]	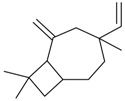
2-Methylene-6,8,8-trimethyl-tricyclo-[5.2.2.0(1,6)]undecan-3-ol (**100**)	*Z. tenuior* [94]	n.f.	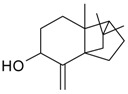
Ar-turmerone (**101**)	*Z. tenuior* [94]	Significant repellent action against *Sitophilus zeamais* and toxic effect against *Spodoptera frugiperda* [413]; insecticidal activities against *Nilaparvata lugens* and *Plutella xylostella* [414] Inhibition of platelet aggregation induced by collagen (IC_50_, 14.4. μM) and arachidonic acid (IC_50_, 43.6. μM) and no effect on PAF and thrombin-induced platelet aggregation on washed rabbit platelets [415]; anti-inflammatory effects through blocking of NF-κB, JNK and p38 MAPK signalling pathways in amyloid β-stimulated microglia [416] Review on activity [417]	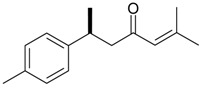

**Table 4:** Miscellaneous substances isolated from Kazakhstan *Ziziphora* species (N.f.—not found).

Name of Compound and ID	Isolation/Detection in Kazakhstan spp. of *Ziziphora* (Percentage Content When Given)	Biological Activity	Chemical Structure
Styrene (**102**)	*Z. tenuior* [94]	n.f.	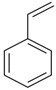
Benzaldehyde (**103**)	*Z. tenuior* [94]	Weak antifungal activity against wood decay fungi [441] Antimicrobial activity against *Listeria monocytogenes* and *Salmonella typhimurium* [442]	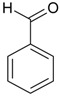
2-Methyl-3-methylbutyl-butanoic acid ester (**104**)	*Z. tenuior* [94]	n.f.	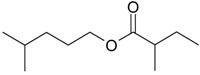
*n*-Amyl isovalerate (**105**)	*Z. tenuior* [94]	n.f.	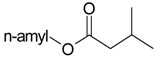
3-Phenyl-2-propenal (syn. cinnamaldehyd) (**106**)	*Z. tenuior* [94]	Antibacterial, antifungal, antidiabetic, anti-inflammatory, antiproliferative activities ([132,443] Inhibition of tyrosin kinase [129]	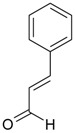
2,4,4,6-Tetramethyl-6-phenyl-1-heptene (**107**)	*Z. tenuior* [94]	n.f.	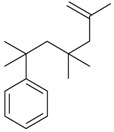
Benzophenone (**108**)	*Z. tenuior* [94]	Photosensitization of lipid peroxidation due to H-abstraction by its long lived triplet state [444]	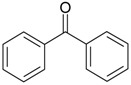
6,10,14-Trimethyl-2-pentadecanone (**109**)	*Z. tenuior* [94]	Could be effective repellent against *Anopheles* species [445]	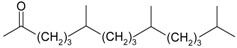
2,6,10,14-Tetramethyl-heptadecane (**110**)	*Z. tenuior* [94]	n.f.	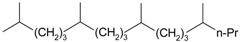
1-octen-3-ol (**111**)	*Z. clinopodioides* [238]	n.f.	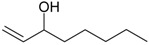
1-octen-3-yl acetate (**112**)	*Z. clinopodioides* subsp. *bungeana* (Juz.) Rech.f. [201]	n.f.	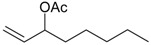
Nonadecane (**113**)	*Z. tenuior* [94]	n.f.	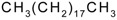
Heneicosane (**114**)	*Z. tenuior* [94]	n.f.	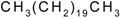
3-octanol (**115**)	*Z. clinopodioides* (0.9%) [184]	n.f.	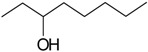
	*Z. tenuior* [94]		
Nonanal (**116**)	*Z. tenuior* [94]	Significant inhibitory effect on mice with diarrhoea induced with castor oil, MgSO_4_ and arachidonic acid [446] Low antifungal activity against fungi of the genus *Colletotrichum* [447]	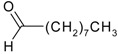
*n*-Decanoic acid (syn. capric acid) (**117**)	*Z. tenuior* [94]	Increasing of total cholesterol level [448]	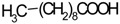
Dodecanoic acid (syn. lauric acid) (**118**)	*Z. tenuior* [94]	Increase in total cholesterol level [448]	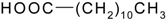
Hexadecanoic acid (syn. palmitic acid) (**119**)	*Z. tenuior* (0.13%–0.31%) [179,202]	Increase in total cholesterol level [448]	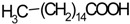
	*Z. tenuior* [94]		
9,12-Octadecadienoic acid (syn. linoleic acid) (**120**)	*Z. tenuior* (0.71%) [187]	Reduction of LDL cholesterol [448] Antibacterial activity against five Gram-positive bacteria [449] and significant activity against rapidly-growing mycobacteria [450]	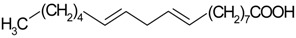
9-Octadecenoic acid (syn. oleic acid) (**121**)	*Z. tenuior* (0.89%) [202]	High concentration may reduce LDL cholesterol [448] Antibacterial activity against three of the five tested Gram-positive bacteria [449]	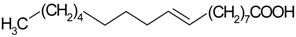
Erigeside B (**122**)	*Z. clinopodioides* [4]	Weak inhibitory activity on NO production stimulated by LPS and IFN-γ in RAW 264.7 cells [4]